# Nematode distributions as spatial null models for macroinvertebrate species richness across environmental gradients: A case from mountain lakes

**DOI:** 10.1002/ece3.2842

**Published:** 2017-03-23

**Authors:** Guillermo de Mendoza, Walter Traunspurger, Alejandro Palomo, Jordi Catalan

**Affiliations:** ^1^ Centre for Advanced Studies of BlanesSpanish National Research Council (CEAB‐CSIC)BlanesSpain; ^2^ Laboratoire GEODE UMR 5602 CNRSUniversité Toulouse‐Jean JaurèsToulouseFrance; ^3^ Department of Animal EcologyFaculty of BiologyUniversity of BielefeldBielefeldGermany; ^4^ Department of Animal Biology, Plant Biology and EcologyFaculty of BiosciencesAutonomous University of BarcelonaCerdanyola del VallèsSpain; ^5^ CREAFCSICEdifici CCampus de Bellaterra (UAB)Cerdanyola del VallèsSpain

**Keywords:** biodiversity, Chironomidae, Insecta, mountain lakes, Nematoda, Oligochaeta, Pyrenees

## Abstract

Nematode species are widely tolerant of environmental conditions and disperse passively. Therefore, the species richness distribution in this group might largely depend on the topological distribution of the habitats and main aerial and aquatic dispersal pathways connecting them. If so, the nematode species richness distributions may serve as null models for evaluating that of other groups more affected by environmental gradients. We investigated this hypothesis in lakes across an altitudinal gradient in the Pyrenees. We compared the altitudinal distribution, environmental tolerance, and species richness, of nematodes with that of three other invertebrate groups collected during the same sampling: oligochaetes, chironomids, and nonchironomid insects. We tested the altitudinal bias in distributions with *t*‐tests and the significance of narrow‐ranging altitudinal distributions with randomizations. We compared results between groups with Fisher's exact tests. We then explored the influence of environmental factors on species assemblages in all groups with redundancy analysis (RDA), using 28 environmental variables. And, finally, we analyzed species richness patterns across altitude with simple linear and quadratic regressions. Nematode species were rarely biased from random distributions (5% of species) in contrast with other groups (35%, 47%, and 50%, respectively). The altitudinal bias most often shifted toward low altitudes (85% of biased species). Nematodes showed a lower portion of narrow‐ranging species than any other group, and differed significantly from nonchironomid insects (10% and 43%, respectively). Environmental variables barely explained nematode assemblages (RDA adjusted R^2^ = 0.02), in contrast with other groups (0.13, 0.19 and 0.24). Despite these substantial differences in the response to environmental factors, species richness across altitude was unimodal, peaking at mid elevations, in all groups. This similarity indicates that the spatial distribution of lakes across altitude is a primary driver of invertebrate richness. Provided that nematodes are ubiquitous, their distribution offers potential null models to investigate species richness across environmental gradients in other ecosystem types and biogeographic regions.

## 1 Introduction

Biodiversity patterns across environmental and spatial gradients have been a focus of ecological research during the last decades (Chase, 2014; Gaston, 2000; Heino, 2011). In the context of biodiversity conservation efforts, understanding the relative contribution of environmental and spatial effects on species richness is particularly relevant in the current situation of biodiversity decline due to increasing habitat loss (Hanski, 2013) and climate change (Parmesan, 2006). On the one hand, biodiversity depends on environmental constraints such as energy availability (Evans, Greenwood, & Gaston, 2005; Waide et al., 1999; Wright, 1983) and ecosystem stability (Connell, 1978; May, 2000). On the other hand, biodiversity depends on habitat area (Connor & McCoy, 2001; Rosenzweig, 2002) and fragmentation (McDonald & Brown, 1992; Thomas et al., 2004). Since the pioneering theory of island biogeography (MacArthur & Wilson, 1967), stochastic colonization and local extinction rates as key driving processes for local species richness have been considered. The metacommunity in a region becomes a key element when considering local species richness in fragmented habitats (Leibold et al., 2004). However, it is not always easy how to disentangle the relative effects of purely spatial factors from environmental influences; particularly, because the biodiversity patterns may be the result of the spatial scale of analysis when studying community assemblages (Chase, 2014; Dümmer, Ristau, & Traunspurger, 2016; Heino, 2011). In this context, comparing patterns of species distribution between different groups of organisms, with varying dispersal capabilities and environmental tolerances, is an approach to empirically evaluate whether environment prevails over spatial factors in determining the biodiversity patterns at a particular spatial scale (Heino, 2013; Heino & Peckarsky, 2014; Mantyka‐Pringle, Martin, & Rhodes, 2013; Urban, Tewksbury, & Sheldon, 2012). Here, we introduce the use of the passively dispersing nematodes in the mountain lakes of the Pyrenees, as a potential null model to evaluate the relative environmental effect on species richness patterns across altitude of other invertebrate groups.

**Figure 1:**
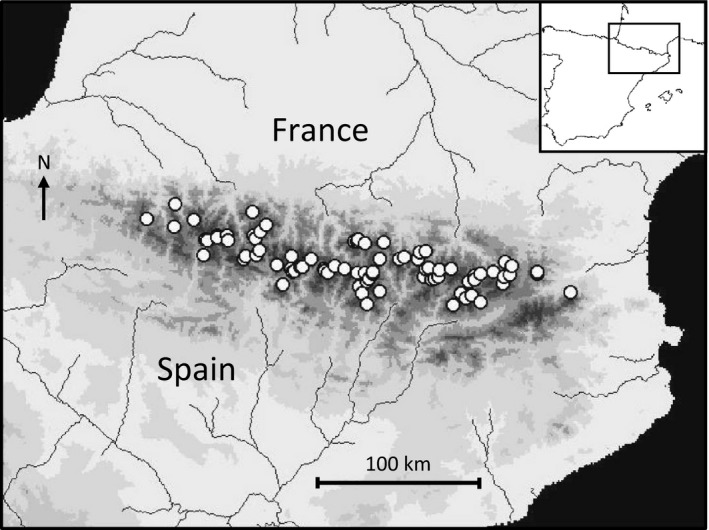
Map with the location of the 82 lakes sampled in this study (white circles)

Nematodes are everywhere. Around a million species of them exist on Earth, and a large proportion (ca. 10,000 of 27,000 described nematode species) are free‐living (Hugot, Baujard, & Morand, 2001), comprising a wide variety of feeding modes (Moens, Traunspurger, & Bergtold, 2006; Traunspurger, 1997; Yeates, Bongers, de Goede, Freckman, & Georgieva, 1993). Previous studies indicate that some nematode species are widely tolerant of environmental conditions. Thus their distribution reflects mostly dispersal, colonization and local extinction stochastic dynamics within a region of fragment habitats. For instance, *Dorylaimus stagnalis* Dujardin and *Plectus cirratus* Bastian are widespread in Africa, where both species have been found in aquatic and terrestrial habitats, the former also even in plankton samples and thermal springs (Jacobs, 1984). Indeed, both species are common also in the high‐altitude lakes of the Alps (Michiels & Traunspurger, 2005). Nematodes can disperse actively at small distances (e.g., entomopathogenic nematodes in their search for a host; Bal, Taylor, & Grewal, 2014), but disperse passively at the scales of mountain lake distributions. These lakes typically occur across large geographic extensions (ca. 15,000 km^2^ in the Pyrenean case, Figure 1) and are often located in different valleys separated by sharp elevations.

In this study, we evaluated whether free‐living nematodes can be used as a spatial null model for investigating the environmental constraints on species richness across altitude in mountain lakes. In the mountains, altitude involves important changes in lake environmental conditions, mainly related to the thermal gradient and related variables. The duration of the ice‐cover period in mountain lakes chiefly depends on altitude (Thompson et al., 2005), with consequences for primary production, for example. Also, the vegetation is less developed at higher altitudes, which affects organic matter and nutrient transfer from the surrounding terrestrial system into the lake (Camarero et al., 2009; Catalan et al., 2006). However, altitude not only implies changes in environmental conditions but also encompasses changes in spatial constraints to dispersal; that is, sharp boundaries in the extremes and shifting degree of lake isolation (density) across elevation. These sharp boundaries, beyond which colonization is not possible, may generate mid‐domain effects (MDE) on biodiversity patterns. Essentially, MDE consist of an increasing overlap of species toward the center of the spatial domain, due to the presence of sharp boundaries at the borders and independently of the influence of species‐environment relationships (Colwell & Lees, 2000; Colwell, Rahbek, & Gotelli, 2004; Pimm & Brown, 2004). Due to the mountain geometry, the sharp boundaries of the spatial domain are governed by the altitudinal gradient. Mountain MDE have been observed for a wide variety of organisms including mammals, birds, plants, and arthropods (Acharya, Sanders, Vijayan, & Chettri, 2011; Brehm, Colwell, & Kluge, 2007; McCain, 2004; Nogués‐Bravo, Araújo, Romdal, & Rahbek, 2008). Furthermore, mountain lakes are not evenly distributed across altitude, and a large proportion of lakes concentrate in the middle of their altitudinal range (Margalef, Campas, Miracle, & Villaseca, 1975; Obertegger, Thaler, & Flaim, 2010), which entails a differential isolation between lakes concerning overland dispersal processes.

We compared the species distribution of nematodes across 82 lakes in the Pyrenees, ranging in altitude from 1620 to 2990 m a.s.l. (de Mendoza & Catalan, 2010), with other invertebrate groups, also collected during the same survey, such as oligochaetes and insects, for which the altitudinal environmental gradient is highly relevant (Collado & de Mendoza, 2009; de Mendoza & Catalan, 2010). Because the altitudinal environmental gradient strongly affects oligochaete and insect species distributions, and previous studies indicate that some nematode species are widely tolerant of environmental changes (Jacobs, 1984; Michiels & Traunspurger, 2005), the environmental tolerance across altitude was expected to be higher for nematodes than for the other invertebrate groups. If this were so, any similarity of species richness patterns between nematodes and other groups could be interpreted as a consequence of similar spatial constraints in the metacommunity dynamics, rather than similar species‐environment relationships. Therefore, we specifically hypothesized (i) that nematodes are scarcely affected by the altitudinal environmental changes compared to other invertebrate groups; and, (ii) that, despite the difference in environmental influence, similarities in species richness patterns between nematodes and other groups indicate that spatial constraints play a major role governing species richness distributions across altitude.

## 2 Methods

### 2.1 Lake selection and sampling

A survey of 82 lakes (Figure 1) representative of the altitudinal and lithological variability in the Pyrenees was performed in summer 2000 (de Mendoza & Catalan, 2010). The lakes were selected based on altitudinal and lithological gradients that determine most of the physical (Thompson, Ventura, & Camarero, 2009) and chemical variability (Camarero et al., 2009; Catalan, Ballesteros, Gacia, Palau, & Camarero, 1993) in mountain lakes. Lakes at the geographic extremes were included to consider the boundaries of the lake district area, and lakes of different size were also chosen within each altitude‐lithology category, wherever possible. The surveyed lakes range from 1,620 to 2,990 m a.s.l. in altitude, and from 0.24 to 53.19 ha in size. Macroinvertebrate sampling was performed in the littoral zone of the lakes at 80 cm depth, which was assumed save to avoid the highly disturbing effects of freezing periods on littoral benthic communities of mountain lakes. The kick‐sampling method of Frost, Huni, and Kershaw (1971) was used with a pond net of 100 μm mesh size, although a 250 μm mesh‐size sieve was eventually used in the laboratory. Sampling was performed at five sampling points per lake of ca. 1 m^2^ each, and during one minute each. Sampling points were selected so as to cover all the habitat types present in each lake. The number of sampling points assigned to each habitat type was weighed by the habitat proportion in the entire littoral zone of each lake, which was assessed by a previous in situ exploration of the whole lake perimeter by several observers. In each sampling area, any large stone present was turned over and brushed in the net, so as to collect invertebrates associated with large stones which would be difficult to collect with the kick‐sampling method. Samples were amalgamated in a unique sample per lake (i.e., 82 samples overall), comprising 5 m^2^ of benthic habitats (weighted by their relative dominance along the littoral zone) and 5 min of sampling effort, in all survey lakes.

### 2.2 Taxonomic determination and groups considered for comparison

All macroinvertebrates collected were sorted according to broad taxonomic groups, being chironomids (Diptera: Chironomidae, ca. 31,000 individuals) and oligochaetes (Oligochaeta, ca. 25,000) the most abundant groups (de Mendoza & Catalan, 2010). Insects other than chironomids were also common (ca. 3,400 individuals), and, therefore, these three groups (oligochaetes, chironomids, and nonchironomid insects) were used for comparing results with that of Nematoda. All nematodes found (ca. 4,000 individuals) were mounted on slides with glycerine, the preparations sealed with paraffin, and species determined under the microscope following mainly Abebe, Andrássy, and Traunspurger (2006). Taxonomic determination of oligochaetes was performed in a previous study (Collado & de Mendoza, 2009), as well as that of some insect groups such as Coleoptera (de Mendoza, Rico, & Catalan, 2012) and Trichoptera (de Mendoza, Ventura, & Catalan, 2015). Chironomids were determined as part of a separate manuscript in preparation (G. de Mendoza, M. Rieradevall and J. Catalan, *unpublished manuscript*). Details on the taxonomic references used for all groups can be found in Appendix S1. The complete species list, with additional information on the altitudinal distribution of species, can be found in Appendix S2.

### 2.3 Environmental variables

Twenty‐eight environmental variables describing the physical and chemical environment, general lake trophic status, littoral substrate and some biotic conditions were measured or determined in the field, or from samples taken at the time of sampling (Catalan, Curtis, & Kernan, 2009), namely: lake area; lake depth; conductivity; pH; total nitrogen (TN); total phosphorus (TP); dissolved organic carbon (DOC); dissolved silica; ammonium; calcium; magnesium; sodium; potassium; sulfate; nitrate; chloride; acid neutralizing capacity (ANC); surface water temperature; organic matter in deep sediment (estimated as loss on ignition, LOI, which is a surrogate of the general trophic status of the lake); chlorophyll‐*a* (Chl‐*a*); bacteria as carbon biomass in plankton samples; substrate granulometry as mean relative abundance of “rocks,” “stones,” “gravel,” and “fine substrate” (estimated by an in situ exploration of the lake littoral zone by several observers independently); macrophyte dominance; and fish occurrence classified as “Salmonidae” and “*Phoxinus,*” to refer to any *Salmo*,* Salvelinus* or *Oncorhynchus,* and *Phoxinus* species, respectively. Samples for all variables were collected (temperature directly measured) at the lake outlet, except for LOI, collected in the deep sediments, and Chl‐*a* and bacteria biomass, collected at a depth of 1.5‐fold the Secchi disk depth, usually corresponding to the deep chlorophyll‐*a* maximum (Catalan et al., 2002). The analytical methods are described in Ventura et al. (2000), except LOI, determined according to Heiri, Lotter, & Lemcke (2001), and bacteria biomass, determined following Straškrabová et al. (1999). The classification of fish occurrence into the two nominal categories (“Salmonidae” and “*Phoxinus”*) was obtained from Miró & Ventura (2013, 2015). Lake area was determined using orthophoto maps and Geographical Information Systems, and lake depth was measured in the field with a portable echo sounder.

### 2.4 Altitudinal distributions

We first visualized, regarding presence/absence data, the altitudinal distribution of each species present in at least four lakes by using box plots: 20 nematodes, 23 oligochaetes, 36 chironomids, and 14 nonchironomid insects. The mean of the altitudinal distribution of each species was registered, and the difference between the highest and the lowest mean within each taxonomic group was annotated. Then, we tested the bias in the altitudinal distribution (presence/absence data) with two‐tailed Student's *t*‐tests (equal variances not assumed) (Zar, 1984), which aimed to compare altitude values between lakes with, and without, the species under analysis. After that, the significance of narrow‐ranging species across altitude was tested by considering first the null expectancy for the altitudinal range of each species, based on its frequency of occurrence. Thus, we defined the altitudinal range expectancy for a species present in *k* lakes as the result of randomly choosing *k* altitude values, without replacement, from those of the lake survey. The confidence intervals for this expectancy were obtained by repeating the procedure 9,999 times using R code (R Core Team 2013). We eventually compared results on altitudinal bias and narrow‐ranging distributions among groups (i.e., the portion of species within each group that was significant in the respective tests) using Fisher's exact tests on contingency tables (Zar, 1984).

### 2.5 Multivariate analysis with species and environmental variables

We used redundancy analysis (RDA) to assess the relationship between environmental factors and species distributions. We transformed raw species data to obtain a Hellinger distance ordination, following Legendre & Gallagher (2001), so as to minimize the influence of zero values in the canonical ordination. Environmental variables departing from normality in a Kolmogorov–Smirnov (KS) goodness‐of‐fit test (Zar, 1984) were previously log‐transformed, except habitat variables (expressed as a percentage) and pH (which is already on a logarithmic scale). Logarithmic transformation was performed as log (x + 1), in order to avoid zeros which do not permit such conversion; for some variables the zeros and negative values (ANC) were transformed into a very small positive number, one order of magnitude below the lowest positive value measured (i.e., 0.001 for K^+^, 0.01 for DOC and NO_3_^−^, and 0.1 for ANC). Environmental variables were included in the RDAs following a forward selection procedure in which the double‐stopping criterion of Blanchet, Legendre, & Borcard (2008) was applied, using the R libraries *vegan* (Oksanen et al., 2013) and *packfor* (Dray, Legendre, & Blanchet, 2013). After performing RDA in each taxonomic group, altitude was fitted onto the RDA ordination using the “envfit” function of the *vegan* package and the correlation (squared correlation coefficient) was tested using 9,999 permutations.

### 2.6 Species richness patterns across altitude

We analyzed species richness patterns across altitude in each taxonomic group with simple linear and quadratic regressions. For each taxonomic group, the most adequate model (i.e., simple linear or quadratic) was defined as the one with the lowest Akaike information criterion value corrected for the number of parameters (AICc), which is highly recommended for low sample numbers (Anderson, 2008). AICc values were computed using the R package *AICcmodavg* (Mazerolle, 2013; R Core Team 2013). The extent of which one model is significantly more adequate than the other was estimated by comparing the two models at a time with chi‐squared tests on a deviance table (R Core Team 2013).

## 3 Results

### 3.1 Altitudinal distributions

**Figure 2:**
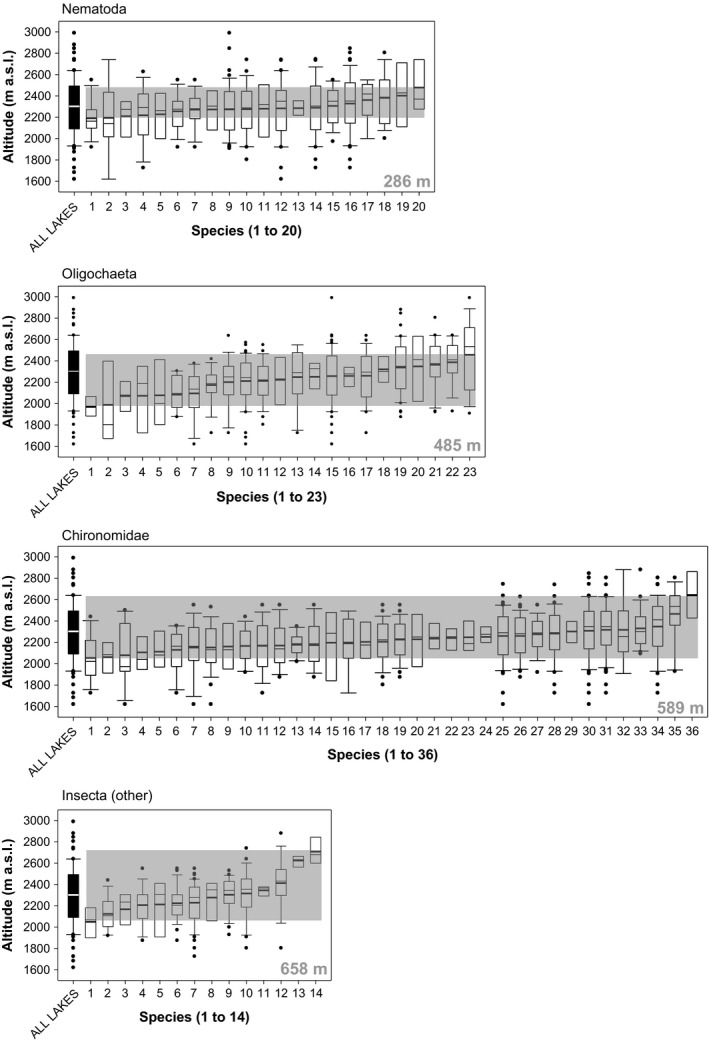
Altitudinal distribution of nematode species, compared to that of oligochaetes, chironomid insects, and nonchironomid insects, also collected during sampling (82 lakes, ranging from 1,620 to 2,990 m a.s.l.). Species are arranged, from left to right, by increasing means of the altitudinal distributions (short thick horizontal bar inside each box plot). The gray band in each diagram defines the altitudinal range (number given in gray color) between the lowest and the highest mean among the species within each group. Only species present in at least four lakes are considered. Numbers are assigned to each species as indicated in Appendix S2

The occurrence of nematode species increased around midaltitudes and tend to segregate less across altitude than the species in the other groups (Figure 2). The mean altitudinal values of the nematode species distributions varied between 2,192 m a.s.l. (*Anatonchus dolichurus*) and 2,478 m a.s.l. (*Eudorylaimus similis*), which implies a difference of 286 m. In the other taxonomic groups considered, the value was higher: 485 m for oligochaetes, 589 m for chironomids, and 658 m for nonchironomid insects. These results indicate that nematode species are far less segregated across the altitudinal gradient than the other groups.

**Figure 3:**
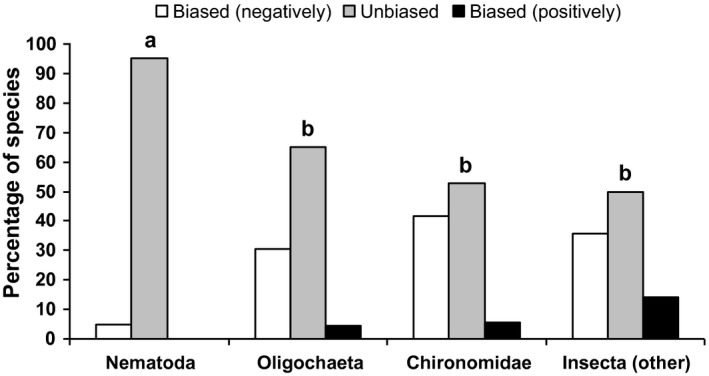
Portion of species with and without altitudinal bias, within each taxonomic group, considering only species present in at least four lakes. Species with altitudinal biased are defined as significant (*p *<* *.05) in a two‐tailed Student's *t*‐test (equal variances not assumed) comparing altitude values between lakes with, and without, that species. Letters (a, b) define groups significantly different in the portion of species with altitudinal bias (*p *<* *.05, Fisher's exact test, biased species in Appendix S2)

Nematode species were rarely biased in their altitudinal distribution in terms of presence/absence data (5% of species), differing significantly (Fisher's exact test, *p *<* *.05) from all other groups: 35%, 47%, and 50% for oligochaetes, chironomids, and nonchironomid insects, respectively (Figure 3). The altitudinal bias considering all taxonomic groups together often deviated toward the lowest altitudes (85% of all biased distributions).

**Figure 4:**
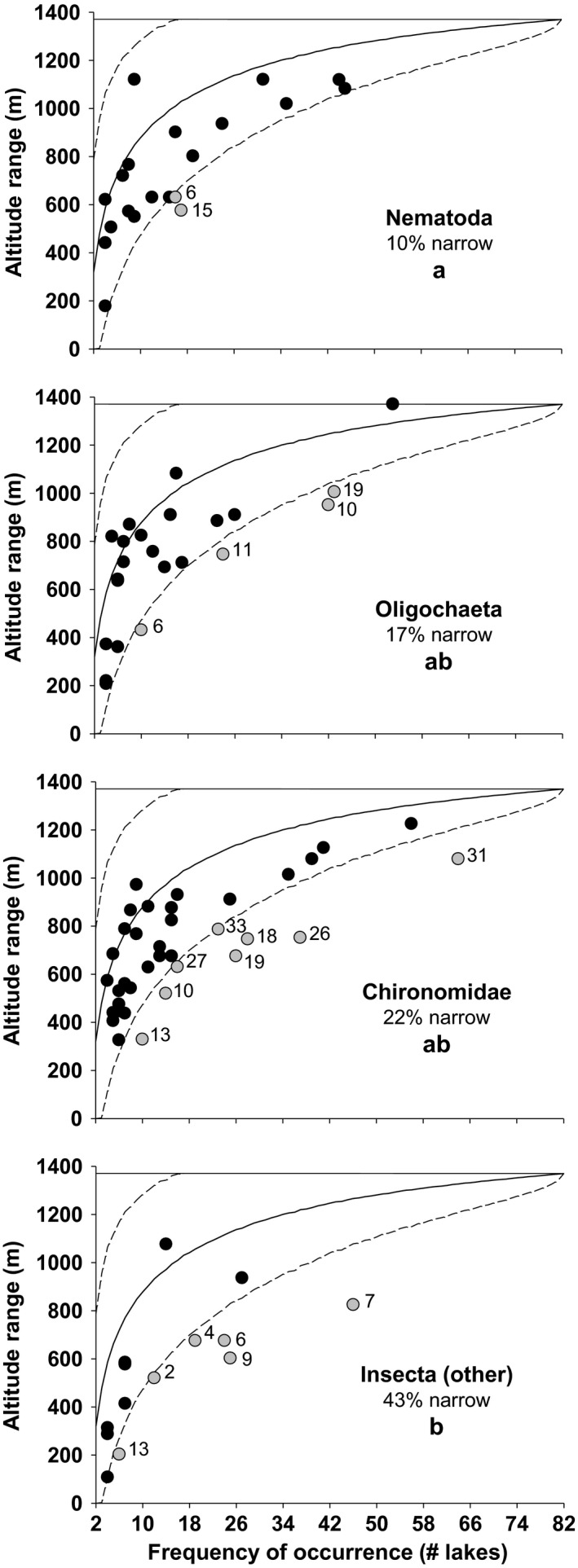
Altitudinal range of species (max. 1,370 m) against their frequency of occurrence (max. 82 lakes), considering only species present in at least four lakes, by groups. Circles are species, represented in gray when narrow‐ranging (numbers are those of Figure 2, species identity in Appendix S2). Narrow‐ranging species are defined by computing 95% confidence intervals (dashed curves) for altitudinal range against the frequency of occurrence (9,999 randomizations of altitude values for each frequency). Letters (a, b) define groups significantly different (*p *<* *.05, Fisher's exact test)

Nematodes showed the lowest portion of narrow‐ranging species (10%), followed by oligochaetes (17%), chironomids (22%) and nonchironomid insects (43%). Fisher's exact tests showed that nematodes differed significantly (*p *<* *.05) from nonchironomid insects in the portion of narrow‐ranging species they hold (Figure 4).

### 3.2 Environmental drivers of species assemblages

**Figure 5:**
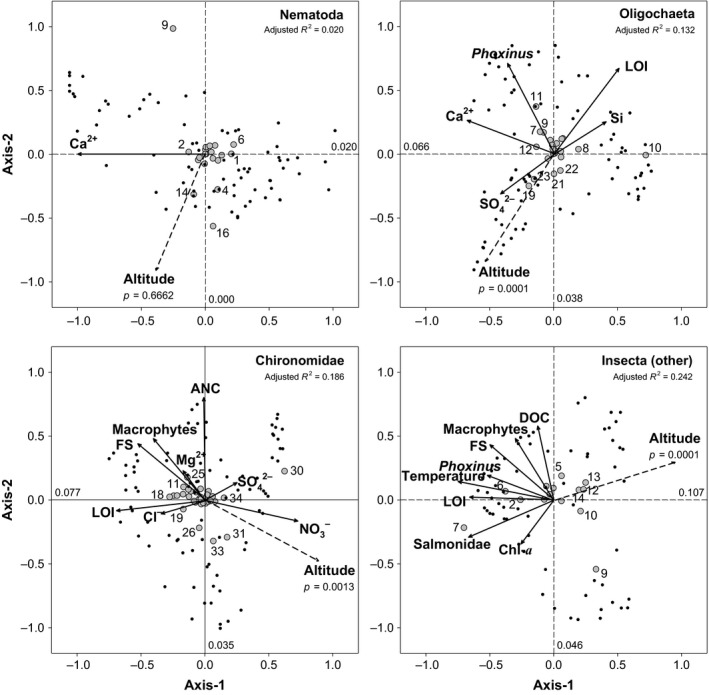
Redundancy analysis (RDA) biplots with species and environmental variables, compared among groups. Altitude was fitted after performing the RDA between species and environmental variables and represented as a short‐dash arrow (*p*‐values refer to the significance of the squared correlation coefficient between altitude and the RDA ordination). The Hellinger distance was used in the ordination, and only species present in at least four lakes (gray circles) were considered. Cumulative adjusted R^2^ values are shown in each RDA, as well as that attributable to each of the first two RDA axes. Information on forward selection of variables is given in Table 1. The location of the species most conspicuously segregating in each biplot is indicated with their associated numbers depicted in Figure 2 (species names in Appendix S2)

Redundancy analysis (RDA) showed that the influence of environmental factors on nematode species assemblages was much lower than in the other groups (Table 1, Figure 5). Forward selected environmental variables, indicating a minimum model of environmental influence, provided adjusted R^2^ values of 0.132, 0.186, and 0.242, for oligochaetes, chironomids, and nonchironomid insects, respectively, and only 0.020 for nematodes. Among all environmental variables considered, only calcium concentration appeared as a significant variable in explaining nematode assemblages, with a very low explanatory capacity, not correlated with altitude (*p *=* *.6662). In contrast, between five and eight environmental variables (related to the thermal, chemical, productivity, and habitat variability of lakes, as well as to the presence of fish) were driving species assemblages in the other groups (Table 1, Figure 5), with an overall explanatory capacity significantly correlated with altitude, from *p *=* *.0013 in chironomids, to *p *=* *.0001 in nonchironomid insects and oligochaetes (Figure 5).

**Table 1:** Environmental variable selection in redundancy analysis (RDA) with Hellinger distance ordination

Nematoda			Oligochaeta			Chironomidae			Insecta (other)		
Variable	*p*	AdjR^2^	Variable	*p*	AdjR^2^	Variable	*p*	AdjR^2^	Variable	*p*	AdjR^2^
Ca^2+^	.0058	**0.020**	Ca^2+^	.0001	0.044	NO_3_^−^	.0001	0.056	Temp.	.0001	0.084
			LOI	.0001	0.080	ANC	.0002	0.084	Salmonid	.0006	0.119
			*Phoxinus*	.0021	0.101	Mg^2+^	.0002	0.113	FS	.0028	0.145
			Si	.0201	0.114	FS	.0011	0.134	Macrph.	.0036	0.167
			SO_4_^2−^	.0028	**0.132**	SO_4_^2−^	.0022	0.154	LOI	.0043	0.188
						LOI	.0115	0.166	DOC	.0069	0.208
						Cl^−^	.0166	0.177	Chl‐*a*	.0098	0.227
						Macrph.	.0254	**0.186**	*Phoxinus*	.0163	**0.242**

Only species present in at least four lakes were considered. The environmental variables (*n *=* *28) were tested in each taxonomic group, following forward selection (*p *<* *.05 after 9,999 Monte Carlo permutations), and the double stopping criterion of Blanchet et al. (2008). AdjR^2^, adjusted R^2^ values after subsequent addition of significant environmental variables (final value highlighted in boldface); LOI, organic matter in deep sediments (loss on ignition); ANC, acid neutralizing capacity; FS, fine substrate dominance in the littoral zone; Macrph., macrophyte dominance in the littoral zone, Temp., temperature; DOC, dissolved organic carbon.

### 3.3 Species richness patterns

**Figure 6:**
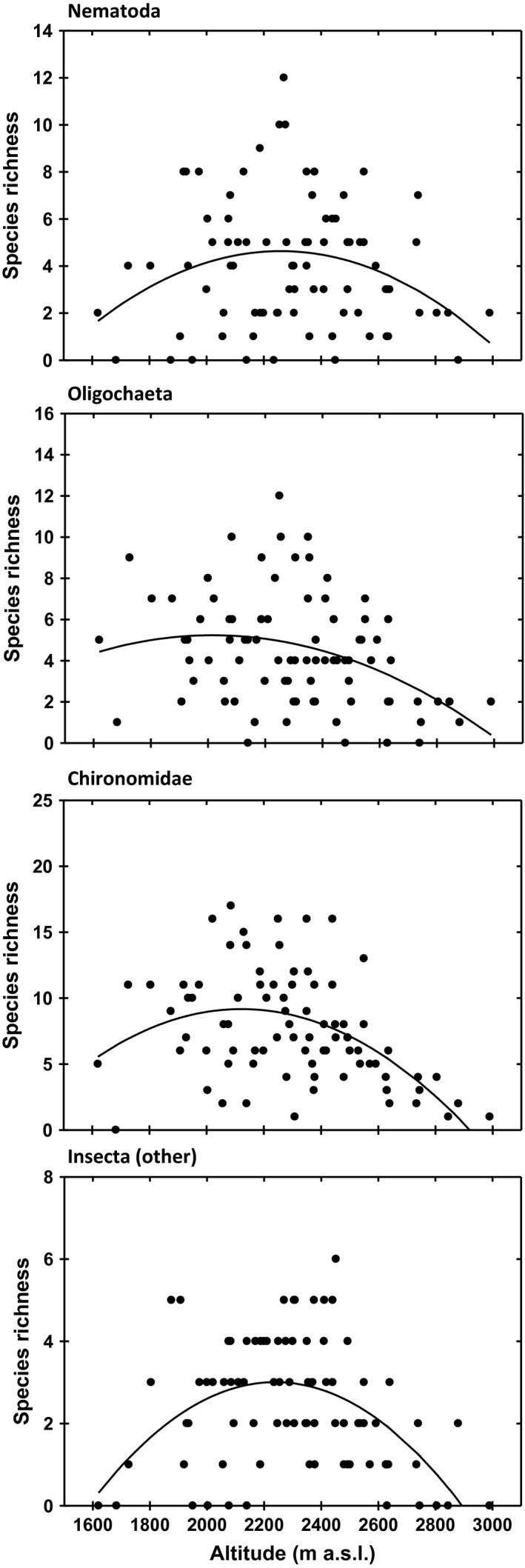
Species richness patterns across altitude using only species present in at least four lakes (as with the other statistical analysis), for each of the taxonomic groups considered. The quadratic regression model was selected over the simple linear model, in all cases (see Table 2). The same results were obtained when using all species (see Figure S1)

The quadratic regression against altitude was favored in front of the simple linear regression in all groups (Table 2, Figure 6). For both nematodes and nonchironomid insects, the simple linear regression was in fact not significant (Table 2). In these two groups, the unimodal relationship is especially clear, and the maximal richness occurred around the same altitude values, 2,255 m a.s.l. for nematodes, and 2,235 m a.s.l. for nonchironomid insects (Figure 6).

**Table 2:** Simple linear and quadratic regression statistics of species richness against altitude for each taxonomic group, by considering only species present in at least four lakes as in all the other statistical analysis

Taxonomic group and model	R^2^	Adj. R^2^	*p*‐value	AICc
Nematodes (*S* = 20)				
Simple linear	0.0035	−0.0089	.5951	402.98
Quadratic	0.0828	0.0595	.0330^a^	**398.40**
Oligochaetes (*S* = 23)				
Simple linear	0.0916	0.0803	.0057^b^	392.10
Quadratic	0.1320	0.1101	.0037^b^	**390.58**
Chironomids (*S* = 36)				
Simple linear	0.1202	0.1092	.0014^b^	460.13
Quadratic	0.2556	0.2367	<.0001^c^	**448.64**
Insecta (other) (*S* = 14)				
Simple linear	0.0222	0.0100	.1813	310.31
Quadratic	0.2462	0.2271	<.0001^c^	**291.19**

Essentially identical results were obtained when considering all species (see Table S1). For each model, R^2^, adjusted R^2^, *p*‐values, and AICc values are shown. The best model (simple linear or quadratic) is defined as the one with the lowest AICc value (highlighted in boldface).

a *p *<* *.05.

b *p *<* *.01.

c *p *<* *.001.

## 4 Discussion

Our results indicate that the nematode species of the mountain lakes of the Pyrenees, unlike other invertebrates, are widely distributed across the altitudinal gradient, and do not show any altitudinal bias in species distributions (Figures 2, 3, and 4). Nematode species do not respond to altitudinal environmental factors, in contrast to the other groups analyzed, as indicated by the explained variance by the environment (Table 1, Figure 5), which was higher in the other groups (oligochaetes 0.13, chironomids 0.19 and nonchironomid insects 0.24) than in nematodes (0.02). However, the comparison of species richness patterns among the different invertebrate groups always reveals a unimodal pattern, where species richness peaks at mid elevations (Table 2, Figure 6). As nematode richness is unlikely to be driven by environmental factors, we must conclude that species richness patterns in mountain lakes, for both nematodes and macroinvertebrates, are mainly driven by factors other than environmental forcing. The similarity between the species richness distribution of nematodes and the other groups indicate that the spatial distribution of lakes across altitude is a major driver of invertebrate richness in mountain lake districts, beyond environmental constraints.

Although environmental factors do not condition nematode assemblages in mountain lakes, the species richness shows the same unimodal pattern with altitude than the other groups. Similar species richness patterns across altitude have been found for benthic invertebrates in other lake districts, such as the Alps (Füreder, Ettinger, Boggero, Thaler, & Thies, 2006) and the Finnish Lapland (Nyman, Korhola, & Brooks, 2005). As discussed in a previous analysis of this dataset (de Mendoza & Catalan, 2010), one possibility is to interpret these patterns regarding the stochastic constraints on the spatial distribution of the lakes. Stochastic constraints will affect biodiversity patterns independently of the group of organisms considered and may include isolation (MacArthur & Wilson, 1967), biogeographic history (Cornell & Lawton, 1992), geometric constraints (Colwell & Lees, 2000), and metacommunity dynamics (Leibold et al., 2004). In fact, mountain lakes are typically unevenly distributed across altitude (Margalef et al., 1975; Obertegger et al., 2010), with more lakes concentrating at mid elevations, which implies reduced isolation as geographic distances between lakes decrease. Likewise, mid‐domain effects derived from geometric constraints (Colwell & Lees, 2000) are also likely to reduce biodiversity in mountain lakes at the extremes of the altitudinal gradient, particularly at the highest altitudes where hard boundaries are unavoidable (i.e., no colonization is possible beyond the highest lakes).

Our results indicate that the spatial stochastic constraints associated with altitude are more relevant than the environmental gradients, independently of the invertebrate group considered; although it is hard to untangle which of these two spatial constraints (i.e., isolation and mid‐domain effects) is more relevant. This conclusion would have been tough to obtain without the use of nematodes as a reference. In this regard, the altitudinal distribution of nematode species richness can be considered as an approximation to a null model for the species richness patterns across altitude of other mountain lake invertebrates (i.e., a model of species richness without environmental influence). In community ecology, a null model is typically defined through randomizations, so as to explore all the possible outcomes (and their associated probabilities) of a response variable, thus as a form of hypothesis testing (Gotelli & Graves, 1996). For example, in the context of comparing species richness values between samples or between sets of samples, rarefaction is a widely used randomization method to know how many species should be expected if fewer individuals, or fewer samples, had been collected (Gotelli & Colwell, 2001). Likewise, in our study, we used randomizations to define “narrow‐ranging species” across the altitudinal gradient (Figure 4). In this case, the null expectancy for the altitudinal range of each species was obtained by computing all possible combinations of *k* altitude values for a given species present in *k* lakes. However, a null model does not necessarily mean “nothing happening”; rather, it can be more generally defined as the outcome of a process or change that would be expected when the mechanism under investigation, which might underlie such change or process, is not acting (Gotelli & Graves, 1996). In this sense, the species richness distribution across altitude of the nematodes of the mountain lakes of the Pyrenees represents a good null model of the influence of environmental factors underlying the species richness distribution of other invertebrates, across the same altitudinal gradient. This nematode's property is simply because the cause of interest in this case (i.e., the effect of environmental factors on species richness across altitude) is barely acting for nematodes. Thus making them a good approximation of the species richness expectancy across altitude in the absence of environmental forcing but submitted to the topological dispersal constraints of the mountains.

Free‐living nematode species inhabiting lakes are poor active dispersers, as indicated also by a recent population genetic study (Ristau, Steinfartz, & Traunspurger, 2013). Passive dispersal may be useful to test nematodes as a null model in front of actively dispersing organisms because it enables to investigate to what extent the observed spatial patterns of richness may be related to stochastic spatial constraints, rather than to the environmental filtering acting upon actively dispersing organisms that select particular environmental conditions. Nematodes disperse mainly passively, for example, by taking advantage of the wind, either as a “dauer” resistance larval form or as eggs (Perry & Moens, 2011; Ptatscheck & Traunspurger, 2015). Also, some species are phoretic, that is, they actively seek for dispersal vectors to attach for a limited time, although this interaction has become parasitic in some cases (Athias‐Binche & Morand, 1993). Phoretic relationships are best known between Rhabditida nematodes and flying insects (Bongers, 1999). Although the Rhabditida were not collected in this survey, phoretic relationships with insects have also been described for other nematodes (Bongers, 1999; Poinar, 1975). Furthermore, nematodes can also be transported by birds in their gut contents (Frisch, Green, & Figuerola, 2007), which indicates the importance of digestive‐resistant forms of nematodes, as suggested by Jacobs (1984). In fact, the most likely hypothesis for the presence of cosmopolitan freshwater nematode species in the Galápagos Islands is through very occasional bird‐mediated transport (Abebe & Coomans, 1995; Muschiol & Traunspurger, 2009). Overall, even if carried by active flyers, dispersal dynamics is, from the nematode perspective, passive, and fundamentally stochastic, which is important to understand the relevance of spatial factors in shaping biodiversity patterns.

Interestingly, nematodes are ubiquitous and present in a broad range of environmental conditions, as shown here by the wide altitudinal distributions of most species, as well as by previous studies emphasizing the wide variety of habitats where some species can be found (e.g., *Dorylaimus stagnalis* and *Plectus cirratus*; Jacobs, 1984; Michiels & Traunspurger, 2005). Therefore, we suggest that the potential of nematode species richness distribution as a null model for the species richness of other invertebrates could be tested across other gradients, biogeographic regions, and ecosystem types, in future research comparisons between different organismal groups.

All in all, nematodes are not neutral to any disturbance, and they can certainly be used for assessing the effects of pollution on species assemblages (Bongers & Ferris, 1999; Heininger, Höss, Claus, Pelzer, & Traunspurger, 2007; Höss, Traunspurger, & Zullini, 2006; Höss et al., 2011, 2017; López‐Doval et al., 2010; Warwick & Clarke, 1998). Feeding habit, generation time, and sensitivity to environmental disturbance allow the designation of functional feeding guilds of different nematode taxa with similar response characteristics (Bongers & Ferris, 1999; Ferris & Bongers, 2006). However, nematodes have variable responses to stress factors; some species are extremely sensitive to pollutants and others extremely tolerant (Faupel & Traunspurger, 2012; Ferris, Bongers, & De Goede, 2004; Höss et al., 2011; Korthals et al., 1996; Tenuta & Ferris, 2004). Also, being nematodes low‐mobility organisms, the use of nematodes as bioindicators is often based on the different colonization capabilities of the nematode families. A given system will show impoverished nematode communities depending on the time lapse after undergoing severe disturbance (Bongers, 1999; Bongers & Ferris, 1999; Höss et al., 2011, 2017).

When no strong environmental disturbance takes place (e.g., pollution episodes), resource availability could favor the persistence of many nematode species. In this context, mountain lakes are stable environments, with generally low amounts of organic matter owing to their low productivity, thus with resource availability playing a minor role governing nematode assemblages. Nematode species richness patterns in lakes with varying productivity have been only reported when large productivity gradients have been considered, including oligotrophic, mesotrophic, and eutrophic lakes (Ristau & Traunspurger, 2011). Even in this situation, the result is not always the same, as observed in 17 Swedish lakes where species richness was highly variable within all the lake trophic state categories considered (Peters & Traunspurger, 2005). Likewise, no relationship was found between nematode species richness and the trophic state of 25 German lakes (Traunspurger, Michiels, & Abebe, 2006). In any case, wide productivity gradients certainly do not apply to the Pyrenean lakes, nor to the mountain lakes in general, in which the oligotrophic state applies in the vast majority of cases. The abundance of submerged vegetation may increase nematode diversity in shallow lakes (Wu & Liang, 1999). This situation is again unlikely for the Pyrenean lakes because macrophyte occurrence is restricted to low altitudes (Gacia et al., 1994; de Mendoza & Catalan, 2010) with limited coverage across the littoral zone (i.e., usually less than 25% of the littoral zone). Therefore, although nematodes can respond to dramatic environmental changes under some circumstances justifying their use in environmental monitoring protocols (e.g., Bongers, 1999; Bongers & Ferris, 1999; Höss et al., 2011, 2017), this is certainly not the case of nematodes in mountain lakes across the altitudinal environmental gradient.

The question of why nematodes in mountain lakes are less sensitive to the environmental change across altitude than other invertebrates analyzed has no clear answer. One possible argument is that nematodes are very rich in species, implying a broad spectrum of responses to environmental conditions, ranging from elevated sensitivity to high resistance, the latter including species that are the last to surrender as environmental conditions deteriorate (Wilson & Kakouli‐Duarte, 2009). These species are possibly far more resistant to environmental changes than most other invertebrates. Nevertheless, our knowledge of nematode ecology and biogeography is still scarce and shows extreme regional bias (Abebe, Decraemer, & De Ley, 2008; Traunspurger et al., 2006). Among the factors contributing to this lack of knowledge, the fact that some free‐living nematodes can only be morphologically recognized as species complexes is noteworthy (Abebe et al., 2008). The issue is highly relevant for nematode biogeography, as the apparent cosmopolitan distribution of free‐living species become nowadays doubtful without genetic support (Ristau et al., 2013; Traunspurger et al., 2006). This apparent cosmopolitanism comprises an astonishingly wide variety of environments, which seems difficult to justify regarding phenotypic plasticity and genetic flexibility (Traunspurger et al., 2006). However, this taxonomical problem minimizes in our study because we are considering strongly shifting environmental conditions (i.e., the altitudinal gradient) in a relatively small geographic area (i.e., the Pyrenees). Thus, the individuals found in Africa and in the Alps of *Dorylaimus stagnalis* and *Plectus cirratus* (Jacobs, 1984; Michiels & Traunspurger, 2005), for example, may not be of the same species if these individuals were analyzed using modern genetic tools, whereas this seems unlikely to occur among the individuals found in the Pyrenees. The assumption that nematode species are widely tolerant to environmental changes does not necessarily imply that nematode species are also cosmopolitan. Although Pennak (1978) referred to nematodes as to “*the most highly adaptable organisms from ecological and physiological stand point,”* the question of why nematodes exhibit such widely tolerant character remains unsolved, primarily because the physiological and genetic flexibility of nematodes is currently far from being understood. An increasing amount of attention is now focusing on reconstructing nematode phylogeny and evolution (Mitreva, Blaxter, Bird, & McCarter, 2005), and these efforts may hopefully provide a satisfactory explanation in the near future concerning the capability of nematodes to survive extremely diverse environmental conditions (Adhikari, Tomasel, Li, Wall, & Adams, 2010). If so, their distribution may offer null models to untangle spatial from environmental constraints in species richness patterns for invertebrates in varied ecosystems and regions.
