# Supplementary material for: Nematode distributions as spatial null models for macroinvertebrate species richness across environmental gradients: A case from mountain lakes
Source: Ecol Evol. 2017 Mar 23;7(9):3016–28. doi: 10.1002/ece3.2842 (PMC5415540; doi:10.1002/ece3.2842)
Supplement: Supplementary file 1 [file ECE3-7-3016-s001.pdf]

Supporting Information to

Nematode distributions as spatial null models for macroinvertebrate species richness across environmental gradients: A case from mountain lakes

Guillermo de Mendoza, Walter Traunspurger, Alejandro Palomo and Jordi Catalan

Ecology and Evolution

Table S1 Species richness regression statistics considering all species in each group.

Figure S1 Species richness patterns across altitude using all species in each group.

Appendix S1 Taxonomic determination of nematodes, oligochaetes and insects.

Appendix S2 Species list, with codes used, and details on altitudinal distribution.

Table S1 Simple linear and quadratic regression statistics of species richness against altitude, for each taxonomic group. All species are taken into account (number of species in each group indicated in brackets), with identical results than those provided when considering only species present in at least four lakes (Table 1). For each model, R^2, adjusted R^2, *P*-values, and AICc values are shown. The best model (simple linear or quadratic) is defined as the one with the lowest AIC value (highlighted in boldface). * *P* < 0.05, ** *P* < 0.01, *** *P* < 0.001.

Taxonomic group and model	R^2	Adj. R^2	*P*-value	AICc
**Nematoda (S = 30)**				
Simple linear	0.0009	–0.0112	0.7852	417.38
Quadratic	0.0821	0.0588	0.0340 *	**412.65**
**Oligochaeta (S = 35)**				
Simple linear	0.1003	0.0890	0.0038 **	399.45
Quadratic	0.1335	0.1116	0.0035 **	**398.57**
**Chironomidae (S = 59)**				
Simple linear	0.1208	0.1098	0.0014 **	474.61
Quadratic	0.2413	0.2221	< 0.0001 ***	**464.73**
**Insecta (other) (S = 42)**				
Simple linear	0.0376	0.0255	0.0811	343.51
Quadratic	0.1863	0.1657	0.0003 ***	**331.95**

Figure S1

Figure S1 Species richness patterns across altitude in each taxonomic group, taking into account all species. The quadratic regression model was selected over the simple linear model, in all cases (see Table S1). The same results were obtained when using only species present in at least four lakes (see Figure 6).

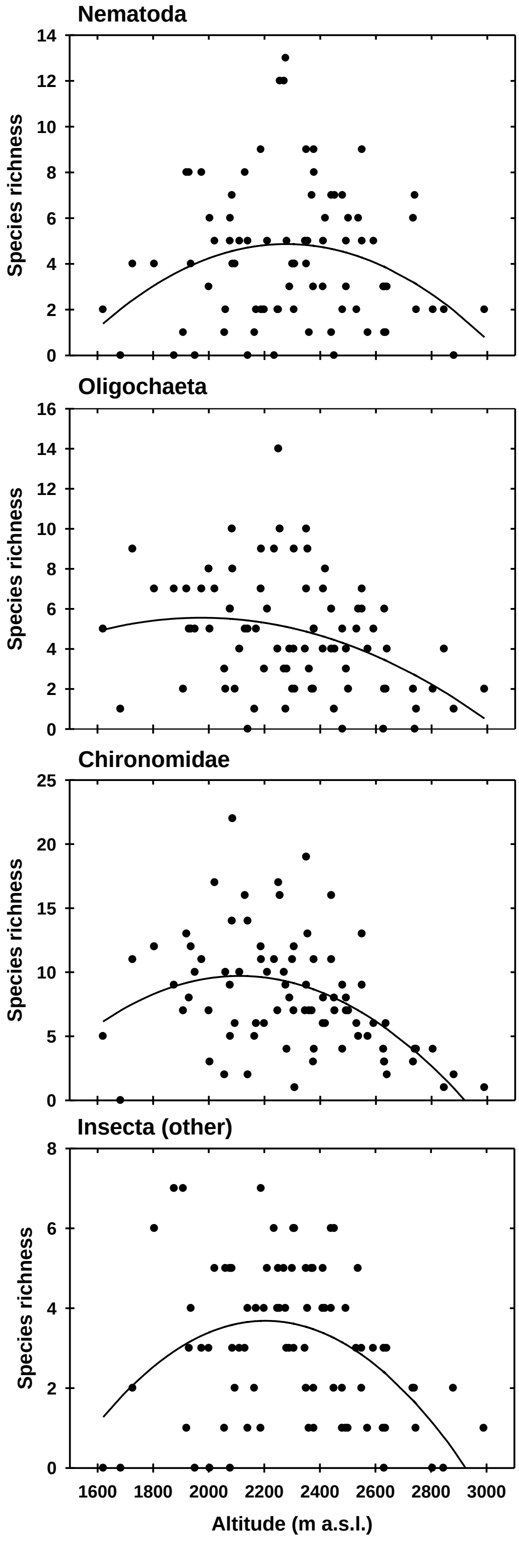

Appendix S1 Taxonomic determination of nematodes, oligochaetes and insects. Nematodes were determined following Abebe et al. (2006). Oligochaetes were previously determined (Collado and de Mendoza 2009), following the taxonomical works of Sperber (1948), Nielsen and Christensen (1959, 1961, 1963), and Brinkhurst and Jamieson (1971). Chironomids were mostly found as larval material, which could be determined under the microscope following mainly Wiederholm (1983) and Rieradevall and Brooks (2001), but also Cranston (1982), with the help of M. Rieradevall (G. de Mendoza, M. Rieradevall and J. Catalan, *unpublished manuscript*). More specific references were also used for particular genera, such as *Corynoneura* and *Psectrocladius* (M. Rieradevall, *personal communication*), *Chironomus* (Laville 1971), *Cricotopus* (Hirvenoja 1973; Nyman et al. 2005), and those of the tribe Tanytarsini (Ekrem 2004; Heiri et al. 2004; M. Rieradevall, *personal communication*). Pupae and pupal exuviae found were in most cases determined to species following mainly Langton (1991), but also Wiederholm (1986), and Wilson (1995) as a starting reference. As in the case of larvae, Hirvenoja (1973) was also used for *Cricotopus* and Ekrem (2004) for *Tanytarsus*. Within non-chironomid insects, species determination also followed specific references; for Coleoptera and Trichoptera, see de Mendoza et al. (2012) and de Mendoza et al. (2015), respectively, and references therein; for Megaloptera, Kaiser (1977) and Elliott (1996) were used; and for other groups the taxonomic determinations were obtained through the assistance of taxonomic experts in their respective fields (Ephemeroptera and Plecoptera, M.A. Puig and N. Ubero-Pascal; Odonata, A. Cordero; Hemiptera, A. Millán; non-chironomid Diptera, E. Bulánková and G. González).

Appendix S1

References

Abebe, E., I. Andrássy, and W. Traunspurger. 2006. Freshwater nematodes: ecology and taxonomy. CABI, Wallingford.

Brinkhurst, R. O., and B. G. M. Jamiesion. 1971. Aquatic Oligochaeta of the world. Oliver & Boyd, Edinburgh.

Collado, R., and G. de Mendoza. 2009. Environmental factors and distribution of littoral oligochaetes in Pyrenean lakes. Advances in Limnology 62:215–244.

Cranston, P. S. 1982. A key to the larvae of the British Orthocladiinae (Chironomidae). Freshwater Biological Association, Ambleside.

de Mendoza, G., E. Rico, and J. Catalan. 2012. Predation by introduced fish constrains the thermal distribution of aquatic Coleoptera in mountain lakes. Freshwater Biology 57:803–814.

de Mendoza, G., M. Ventura, and J. Catalan. 2015. Environmental factors prevail over dispersal constraints in determining the distribution and assembly of Trichoptera species in mountain lakes. Ecology and Evolution 5:2518–2532.

Ekrem, T. 2004. Immature stages of European *Tanytarsus* species I. The *eminulus*-, *gregarius*-, *lugens*-, and *mendax* species groups (Diptera, Chironomidae). Mitteilungen aus dem Museum für Naturkunde in Berlin, Deutsche Entomologische Zeitschrift 51:97–146.

Elliott, J. M. 1996. British freshwater Megaloptera and Neuroptera: a key with ecological notes. Freshwater Biological Association, Ambleside.

Heiri, O., T. Ekrem, and E. Willassen. 2004. Larval head capsules of European *Micropsectra*, *Paratanytarsus* and *Tanytarsus* (Diptera: Chironomidae: Tanytarsini). Version 1.0, August 24, 2004. http://www3.bio.uu.nl/palaeo/Chironomids/Tanytarsini/Intro.htm [Downloaded on March 20, 2007].

Hirvenoja, M. 1973. Revision der Gattung *Cricotopus* van der Wulp und ihrer Verhwandten (Diptera, Chironomidae). Annales Zoologici Fennici 10:1–363.

Kaiser, E. W. 1977. Æg og larver af 6 *Sialis*-arter fra Skandinavien og Finland. Flora Fauna, Arhus 83:65–79.

Langton, P. H. 1991. A key to pupal exuviae of West Palearctic Chironomidae. Privately published by P.H. Langton, 3 St Felix Road, Ramsey Forty Foot, Huntingdon, Cambridgeshire.

Appendix S1

Laville, H. 1971. Recherches sur les chironomides (Diptera) lacustres du Massif de Néouvielle (Hautes-Pyrénées). Première partie: systematique, écologie, phénologie. Annales de Limnologie 7:173–332.

Nielsen, C. O., and B. Christensen. 1959. The Enchytraeidae, critical revision and taxonomy of European species. Natura Jutlandica 8/9:1–160.

Nielsen, C. O., and B. Christensen. 1961. The Enchytraeidae, critical revision and taxonomy of European species. Natura Jutlandica 10 (Suppl. 1):1–23.

Nielsen, C. O., and B. Christensen. 1963. The Enchytraeidae, critical revision and taxonomy of European species. Natura Jutlandica 10 (Suppl. 2):1–19.

Nyman, M., A. Korhola, and S. J. Brooks. 2005. The distribution and diversity of Chironomidae (Insecta: Diptera) in western Finnish Lapland, with special emphasis on shallow lakes. Global Ecology and Biogeography 14:137–153.

Rieradevall, M., and S. J. Brooks. 2001. An identification guide to subfossil Tanypodinae larvae (Insecta: Diptera: Chironomidae) based on cephalic setation. Journal of Paleolimnology 25:81–99.

Sperber, C. 1948. A taxonomical study of the Naididae. Zoologiska Bidrag från Uppsala 28:1–296.

Wiederholm, T. 1983. Chironomidae of the Holarctic region. Keys and diagnoses. Part 1. Larvae. Entomologica Scandinavica Suppl. 19:1–457.

Wiederholm, T. 1986. Chironomidae of the Holarctic region. Keys and diagnoses. Part 2. Pupae. Entomologica Scandinavica Suppl. 28:1–482.

Wilson, R. S. 1995. A practical key to the genera of pupal exuviae of the British Chironomidae (Diptera: Insecta), unpublished revised version, August 1995, of Wilson, R. S. and J. D. McGill, 1982, A practical key to the genera of pupal exuviae of the British Chironomidae (Diptera: Insecta), University of Bristol Printing Office, Bristol.

Appendix S2 Species considered, with the associated numbers ('#') used in the main manuscript (Figures 2, 4 and 5), separately for each taxonomic group. Detailed information is given for the frequency of occurrence of species ('Freq.', referred to as the number of lakes where the species was collected, max. 82); for the altitudinal bias in distributions ('Bias', indicating whether the bias is positive or negative in square brackets when significant, $P < 0.05$ in Figure 3); and for the narrow ranging character of species ('Narrow', indicated by asterisks when significant, $P < 0.05$ in Figure 4). Species found in less than four lakes were not considered (n/c) in the main manuscript, but are used in the Supporting Information analyses of Table S1 and Figure S1. Some individuals were not determined to species-level, but belonged to the same morphotype; these cases were considered as of the same species, except in those instances where one morphotype was confidently representing more than one species, thus being discarded from statistical analysis in both the main manuscript and the Supporting Information (as it was the case of the few taxa determined to family-level). *Micropsetra*, *Tanytarus* and *Paratanytarsus* types were assigned following type descriptions of Heiri et al. (2004). In nematodes, oligochaetes and non-chironomid insects, the taxonomic Order to which each species belongs is indicated. As chironomids are a family of insects in the Order Diptera, in this case, the subfamily or tribe of each species is shown.

Appendix S2

Nematoda					
**Species name**	**Family (Order)**	**#**	**Freq.**	**Bias**	**Narrow**
Ironus longicaudatus de Man	Ironidae (Enoplida)	15	17		*
Ironus tenuicaudatus de Man	Ironidae (Enoplida)	4	16		
Eutobrilus grandipapillatus (Brakenhoff)	Tobrilidae (Triplonchida)	12	31		
Semitobrilus pellucidus (Bastian)	Tobrilidae (Triplonchida)	7	15		
Tobrilus gracilis (Bastian)	Tobrilidae (Triplonchida)	19	8		
Tripyla glomerans Bastian	Tripylidae (Triplonchida)	14	35		
Crocodorylaimus flavomaculatus (Linstow)	Dorylaimidae (Dorylaimida)	2	9		
Dorylaimus cf. *stagnalis* Dujardin	Dorylaimidae (Dorylaimida)	9	45		
Mesodorylaimus sp.1 (*conurus*?)	Dorylaimidae (Dorylaimida)	13	4		
Prodorylaimus cf. *rotundiceps* Loof	Dorylaimidae (Dorylaimida)	8	5		
Paractinolaimus macrolaimus (de Man)	Actinolaimidae (Dorylaimida)	6	16		*
Epidorylaimus consobrinus (de Man)	Qudsianematidae (Dorylaimida)	16	44		
Eudorylaimus similis (de Man)	Qudsianematidae (Dorylaimida)	20	7		
Aporcelaimellus obtusicaudatus (Bastian)	Aporcelaimidae (Dorylaimida)	10	24		
Anatonchus dolichurus (Ditlevsen)	Anatonchidae (Mononchida)	1	12	[-]	
Coomansus zschokkei (Menzel)	Mononchidae (Mononchida)	17	9		
Mononchus truncatus Bastian	Mononchidae (Mononchida)	18	19		
Ethmolaimus cf. *pratensis* de Man	Ethmolaimidae (Chromadorida)	3	4		
Plectus aquatilis Andrássy	Plectidae (Plectida)	5	8		
Plectus cirratus Bastian	Plectidae (Plectida)	11	4		
Discarded (present in less than four lakes)					
Tobrilus sp.2	Tobrilidae (Triplonchida)	-	1	n/c	n/c
Tripyla filicaudata de Man	Tripylidae (Triplonchida)	-	1	n/c	n/c
Ischiodorylaimus cf. *cognatus* Andrássy	Dorylaimidae (Dorylaimida)	-	1	n/c	n/c
Prodorylaimus filiarum Andrássy	Dorylaimidae (Dorylaimida)	-	1	n/c	n/c
Clarkus papillatus (Bastian)	Mononchidae (Mononchida)	-	2	n/c	n/c
Prionchulus cf. *punctatus* (Cobb)	Mononchidae (Mononchida)	-	1	n/c	n/c
Prionchulus muscorum (Dujardin)	Mononchidae (Mononchida)	-	2	n/c	n/c
Achromadora terricola (de Man)	Achromadoridae (Chromadorida)	-	2	n/c	n/c
Monhystera cf. *paludicola* de Man	Monhysteridae (Monhysterida)	-	2	n/c	n/c
Aphanolaimus aquaticus von Daday	Aphanolaimidae (Plectida)	-	2	n/c	n/c
Discarded (not determined to species level)					
Mermithidae (undetermined)	Mermithidae (Mermithida)	-	31	n/c	n/c

Appendix S2

Oligochaeta					
**Species name**	**Family (Order)**	**#**	**Freq.**	**Bias**	**Narrow**
Lumbriculus variegatus (Müller)	Lumbriculidae (Lumbriculida)	17	26		
Stylodrilus heringianus Claparède	Lumbriculidae (Lumbriculida)	21	23		
Bothrioneurum vejdovskyanum Stolc	Naididae (Haplotaxida)	3	4	[-]	
Chaetogaster diaphanus (Gruithuisen)	Naididae (Haplotaxida)	4	7		
Chaetogaster diastrophus (Gruithuisen)	Naididae (Haplotaxida)	5	7		
Limnodrilus hoffmeisteri Claparède	Naididae (Haplotaxida)	20	8		
Nais barbata Müller	Naididae (Haplotaxida)	2	5		
Nais bretscheri Michaelsen	Naididae (Haplotaxida)	9	15		
Nais cf. *communis* Piguet	Naididae (Haplotaxida)	18	6		
Nais pardalis Piguet	Naididae (Haplotaxida)	14	6		
Nais pseudobtusa Piguet	Naididae (Haplotaxida)	7	12	[-]	
Nais simplex Piguet	Naididae (Haplotaxida)	11	24	[-]	*
Nais variabilis Piguet	Naididae (Haplotaxida)	15	53		
Slavina appendiculata (d'Udekem)	Naididae (Haplotaxida)	16	4		
Specaria josinae (Vejdovský)	Naididae (Haplotaxida)	10	42	[-]	*
Spirosperma ferox (Eisen)	Naididae (Haplotaxida)	6	10	[-]	*
Tubifex tubifex (Müller)	Naididae (Haplotaxida)	22	17		
Uncinais uncinata (Ørsted)	Naididae (Haplotaxida)	1	4	[-]	
Vejdovskyella comata (Vejdovský)	Naididae (Haplotaxida)	8	14	[-]	
Cognettia glandulosa (Michaelsen)	Enchytraeidae (Haplotaxida)	19	43		*
Cognettia sphagnetorum (Vejdovský)	Enchytraeidae (Haplotaxida)	23	16	[+]	
Cognettia sp.3	Enchytraeidae (Haplotaxida)	13	10		
Henlea perpusilla Friend	Enchytraeidae (Haplotaxida)	12	6		
<u>Discarded (present in less than four lakes)</u>					
Chaetogaster cristallinus Vejdovský	Naididae (Haplotaxida)	-	2	n/c	n/c
Nais alpina Sperber	Naididae (Haplotaxida)	-	2	n/c	n/c
Pristina aequiseta Bourne	Naididae (Haplotaxida)	-	2	n/c	n/c
Pristina cf. *jenkinae* (Stephenson)	Naididae (Haplotaxida)	-	1	n/c	n/c
Pristina sp.3	Naididae (Haplotaxida)	-	1	n/c	n/c
Buchholzia appendiculata (Buchholz)	Enchytraeidae (Haplotaxida)	-	1	n/c	n/c
Henlea sp.2	Enchytraeidae (Haplotaxida)	-	3	n/c	n/c
Mesenchytraeus armatus (Levinsen)	Enchytraeidae (Haplotaxida)	-	1	n/c	n/c
Fridericia cf. *bulboides* Niels. & Christens.	Enchytraeidae (Haplotaxida)	-	1	n/c	n/c
Fridericia sp.2	Enchytraeidae (Haplotaxida)	-	1	n/c	n/c
Lumbricidae (undetermined)[1]	Lumbricidae (Haplotaxida)	-	1	n/c	n/c
Aeolosoma sp.	Aeolosomatidae (Haplotaxida)	-	2	n/c	n/c
<u>Discarded (not determined to species level)</u>					
Cernosvitoviella spp.	Enchytraeidae (Haplotaxida)	-	49	n/c	n/c
Achaeta spp.	Enchytraeidae (Haplotaxida)	-	20	n/c	n/c

[1] Only one individual found, therefore only one species.

Appendix S2

Chironomidae

**Species name**	**Subfamily / tribe**	**#**	**Freq.**	**Bias**	**Narrow**
Ablabesmyia longistyla Fittkau	Tanypodinae	19	26	[-]	*
Ablabesmyia monilis (Linnaeus)	Tanypodinae	8	25	[-]	
Ablabesmyia phatta (Egger)	Tanypodinae	32	9		
Trissopelopia sp.	Tanypodinae	24	7		
Conchapelopia sp.	Tanypodinae	22	6		
Thienemannimyia sp.	Tanypodinae	5	6	[-]	
Macropelopia gr. *notata*	Tanypodinae	33	23		*
Macropelopia gr. *nebulosa*	Tanypodinae	34	39		
Prodiamesa olivacea (Meigen)	Prodiamesinae	2	5	[-]	
Chaetocladius sp.	Orthocladiinae	17	6		
Corynoneura lacustris-type	Orthocladiinae	26	37		*
Cricotopus pirifer Hirvenoja	Orthocladiinae	3	11	[-]	
Cricotopus gr. *reversus*	Orthocladiinae	21	7		
Cricotopus gr. *sylvestris*	Orthocladiinae	11	15	[-]	
Heterotrissocladius marcidus (Walker)	Orthocladiinae	31	64		*
Metriocnemus gr. *hygropetricus*	Orthocladiinae	15	8		
Paratrichocladius sp.	Orthocladiinae	36	5	[+]	
Psectrocladius gr. *psilopterus*	Orthocladiinae	27	16		*
Psectrocladius gr. *sordidellus*	Orthocladiinae	7	16	[-]	
Synorthocladius semivirens (Kieffer)	Orthocladiinae	28	35		
Thienemannia sp.	Orthocladiinae	9	7		
Chironomus commutatus-type	Chironomini	1	13	[-]	
Cladopelma sp.	Chironomini	4	8	[-]	
Microtendipes gr. pedellus	Chironomini	10	14	[-]	*
Pagastiella orophila (Edwards)	Chironomini	13	10	[-]	*
Paracladopelma sp.	Chironomini	16	9		
Polypedilum nubens-type	Chironomini	14	15	[-]	
Polypedilum albicorne-type	Chironomini	6	11	[-]	
Pseudochironomus prasinatus (Stæger)	Pseudochironomini	23	5		
Cladotanytarsus gr. *mancus*	Tanytarsini	12	13	[-]	
Micropsectra radialis-type	Tanytarsini	35	15	[+]	
Micropsectra C-type	Tanytarsini	20	4		
Nezavrelia A-type *(luteola?)*	Tanytarsini	29	6		
Paratanytarsus austriacus-type	Tanytarsini	30	56		
Paratanytarsis penicillatus-type	Tanytarsini	18	28	[-]	*
Tanytarsus lugens-type	Tanytarsini	25	41		
<u>Discarded (present in less than four lakes)</u>					
Pseudokiefferiella parva (Edwards)	Diamesinae	-	2	n/c	n/c
Brillia sp.	Orthocladiinae	-	1	n/c	n/c
Corynoneura cf. *lobata* Edwards	Orthocladiinae	-	1	n/c	n/c
Cricotopus pulchripes-type	Orthocladiinae	-	3	n/c	n/c
Cricotopus species A	Orthocladiinae	-	1	n/c	n/c
Cricotopus gr. *obnixus*	Orthocladiinae	-	1	n/c	n/c
Heterotanytarsus sp.	Orthocladiinae	-	1	n/c	n/c
Paracladius sp.	Orthocladiinae	-	3	n/c	n/c
Parakiefferiella coronata (Edwards)	Orthocladiinae	-	1	n/c	n/c
Parorthocladius sp.	Orthocladiinae	-	2	n/c	n/c
Psectrocladius (Mesopsectrocladius) sp.	Orthocladiinae	-	2	n/c	n/c
Rheocricotopus sp.	Orthocladiinae	-	1	n/c	n/c
Tvetenia sp.	Orthocladiinae	-	1	n/c	n/c
Dicrotendipes tritomus (Kieffer)	Chironomini	-	3	n/c	n/c
Einfeldia pagana (Meigen)	Chironomini	-	1	n/c	n/c
Parachironomus gr. *arcuatus*	Chironomini	-	1	n/c	n/c
Phaenopsectra sp.	Chironomini	-	2	n/c	n/c
Stictochironomus sp.	Chironomini	-	1	n/c	n/c

Appendix S2

Chironomidae (continued)					
**Species name**	**Subfamily / tribe**	**#**	**Freq.**	**Bias**	**Narrow**
Micropsectra insignilobus-type	Tanytarsini	-	1	n/c	n/c
Neozavrelia B-type	Tanytarsini	-	1	n/c	n/c
Stempellinella (?) sp.	Tanytarsini	-	1	n/c	n/c
Tanytarsus chinyensis-type	Tanytarsini	-	2	n/c	n/c
Tanytarsus nemorosus-type	Tanytarsini	-	2	n/c	n/c
<u>Discarded (not determined to species level)</u>					
Zavrelimyia spp.	Tanypodinae	-	45	n/c	n/c
Procladius spp.	Tanypodinae	-	47	n/c	n/c
Diamesa spp.	Diamesinae	-	4	n/c	n/c
Pseudodiamesa spp.	Diamesinae	-	15	n/c	n/c
Corynoneura scutellata-type	Orthocladiinae	-	43	n/c	n/c
Orthocladius spp.	Orthocladiinae	-	11	n/c	n/c
Psectrocladius octomaculatus-type (spp.)	Orthocladiinae	-	34	n/c	n/c
Chironomus / Einfeldia C-type	Chironomini	-	4	n/c	n/c
Micropsectra aristata-type (spp.)	Tanytarsini	-	35	n/c	n/c
Tanytarsus mendax-type	Tanytarsini	-	16	n/c	n/c

Appendix S2

**Insecta (other)**					
**Species name**	**Family (Order)**	**#**	**Freq.**	**Bias**	**Narrow**
Aeshna sp.	Aeshnidae (Odonata)	1	4	[-]	
Cloëon schoenemundi Bengtsson	Baetidae (Ephemeroptera)	4	19	[-]	*
Capnia vidua Klapálek	Capniidae (Plecoptera)	14	4	[+]	
Arctocorisa carinata (Sahlberg)	Corixidae (Hemiptera)	5	7		
Sialis lutaria (Linnaeus)	Sialidae (Megaloptera)	7	46	[-]	*
Boreonectes cf. *ibericus* (Dutton & Angus)	Dytiscidae (Coleoptera)	8	7		
Oreodytes cf. *sanmarkii* (Sahlberg)	Dytiscidae (Coleoptera)	11	4		
Agabus cf. *bipustulatus* (Linnaeus)	Dytiscidae (Coleoptera)	12	14		
Platambus maculatus (Linnaeus)	Dytiscidae (Coleoptera)	3	7		
Plectrocnemia cf. *laetabilis* McLachlan	Polycentropodidae (Trichoptera)	9	25		*
Polycentropus flavomaculatus (Pictet)	Polycentropodidae (Trichoptera)	6	24	[-]	*
Drusus cf. *rectus* (McLachlan)	Limnephilidae (Trichoptera)	13	6	[+]	*
Annitella cf. *pyrenaea* (Navás)	Limnephilidae (Trichoptera)	10	27		
Mystacides azurea (Linnaeus)	Leptoceridae (Trichoptera)	2	12	[-]	*
Discarded (present in less than four lakes)					
Enallagma cyathigerum (Charp)	Coenagrionidae (Odonata)	-	1	n/c	n/c
Caenis horaria (Linnaeus)	Caenidae (Ephemeroptera)	-	2	n/c	n/c
Ecdyonurus cf. *forcipula* (Pictet)	Heptageniidae (Ephemeroptera)	-	1	n/c	n/c
Electrogena lateralis (Curtis)	Heptageniidae (Ephemeroptera)	-	1	n/c	n/c
Habroleptoides gr. *umbratilis*	Leptophlebiidae (Ephemeroptera)	-	1	n/c	n/c
Siphlonurus lacustris Eaton	Siphlonuridae (Ephemeroptera)	-	1	n/c	n/c
Arcynopteryx compacta (McLachlan)	Perlodidae (Plecoptera)	-	3	n/c	n/c
Perlodes intricata (Pictet)	Perlodidae (Plecoptera)	-	2	n/c	n/c
Siphonoperla torrentium (Pictet)	Chloroperlidae (Plecoptera)	-	3	n/c	n/c
Nemoura cinerea Retzius	Nemouridae (Plecoptera)	-	3	n/c	n/c
Nemoura mortoni Ris	Nemouridae (Plecoptera)	-	1	n/c	n/c
Nemurella pictetii Klapálek	Nemouridae (Plecoptera)	-	1	n/c	n/c
Leuctra leptogaster Aubert	Leuctridae (Plecoptera)	-	2	n/c	n/c
Micronecta poweri (Douglas & Scott)	Corixidae (Hemiptera)	-	1	n/c	n/c
Haliplus cf. *fulvus* (Fabricius)	Haliplidae (Coleoptera)	-	3	n/c	n/c
Helophorus sp.	Helophoridae (Coleoptera)	-	1	n/c	n/c
Elmis sp.	Elmidae (Coleoptera)	-	1	n/c	n/c
Limonia sp.	Limoniidae (Diptera)	-	1	n/c	n/c
Erioptera sp.	Limoniidae (Diptera)	-	1	n/c	n/c
Limnophila sp.	Limoniidae (Diptera)	-	1	n/c	n/c
Simulium sp.	Simuliidae (Diptera)	-	1	n/c	n/c
Chrysops sp.	Tabanidae (Diptera)	-	1	n/c	n/c
Lispe sp.	Muscidae (Diptera)	-	1	n/c	n/c
Agrypnia sp.	Phryganeidae (Trichoptera)	-	1	n/c	n/c
Potamophylax latipennis (Curtis)	Limnephilidae (Trichoptera)	-	3	n/c	n/c
Limnephilus cf. *guadarramicus* Schmid	Limnephilidae (Trichoptera)	-	3	n/c	n/c
Thremma gallicum McLachlan	Limnephilidae (Trichoptera)	-	3	n/c	n/c
Athripsodes aterrimus (Stephens)	Leptoceridae (Trichoptera)	-	1	n/c	n/c
Discarded (not determined to species level)					
Hydroporus spp.[2]	Dytiscidae (Coleoptera)	-	28	n/c	n/c
Ceratopogonidae (undetermined)	Ceratopogonidae (Diptera)	-	6	n/c	n/c

[2] Only one species was found in our lake survey, *H. foveolatus* Heer, but the genus is highly diversified in the Pyrenees (Ribera et al., 1993), and other species are likely present.

Appendix S2

References

Heiri, O., T. Ekrem, and E. Willassen. 2004. Larval head capsules of European *Micropsectra*, *Paratanytarsus* and *Tanytarsus* (Diptera: Chironomidae: Tanytarsini). Version 1.0, August 24, 2004. http://www3.bio.uu.nl/palaeo/Chironomids/Tanytarsini/Intro.htm [Downloaded on March 20, 2007].

Ribera, I., C. Hernando, X. Fresneda, P. Aguilera, G. N. Foster, and S. Bignal. 1993. A preliminary checklist of the Hydradephaga from the Pyrenees. Latissimus 3:6–10.
